# Lacrimal sac bacteriology and susceptibility pattern in infants with congenital nasolacrimal duct obstruction in the 1st year of life: a cross-sectional study

**DOI:** 10.1186/s12887-020-02358-5

**Published:** 2020-10-06

**Authors:** Xiao-Yu Zheng, Bonnie Nga Kwan Choy, Ming-Ming Zhou, Cai-Ping Shi, Zheng-Yan Zhao

**Affiliations:** 1 Department of Ophthalmology, The Children’s Hospital, Zhejiang University School of Medicine, National Clinical Research Center for Child Health, No. 3333 Binsheng Road, Hangzhou, Zhejiang Province PR China 310052; 2 Department of Ophthalmology, LKS Faculty of Medicine, The University of Hong Kong, Hong Kong, China; 3 Department of Clinical Lab, The Children’s Hospital, Zhejiang University School of Medicine, National Clinical Research Center for Child Health, Hangzhou, Zhejiang China; 4 Department of Child Health Care, The Children’s Hospital, Zhejiang University School of Medicine, National Clinical Research Center for Child Health, Hangzhou, Zhejiang China

**Keywords:** Congenital nasolacrimal duct obstruction, Infant, Microbiology, Lacrimal sac, Levofloxacin

## Abstract

**Background:**

Congenital nasolacrimal duct obstruction (CNLDO) is one of the main causes of epiphora in infants, and antibiotics are usually used as a conservative therapy in the first year. Yet, little is known about the bacteriology of the occluded lacrimal drainage system in this group of patients. The aim of this study was to evaluate the microbiology of lacrimal sac (LS) in Chinese children with CNLDO in their first year of life.

**Methods:**

Patients with CNLDO between May 1, 2017 and August 31, 2018 at a tertiary care children’s hospital were enrolled. The study recruited infants who received lacrimal probing under 1 year old, and refluxed discharge from LS was collected. Samples were cultured and susceptibility test was performed for positive culture.

**Results:**

Thirty-two patients with CNLDO were included. The ratio of male to female was 23:9. The mean age was 6.7 ± 2.4 (1.7–12) months. Positive cultures was identified in 87.5% of the sample, and presented 38 strains of bacteria. Mixed infection was identified in 10 (31.3%) children. Gram-positive bacteria accounted for 60.5% of all the strains, with *Streptococcus* (50%) being the most frequent species, whereas *Haemophilus* (21.1%) and *Neisseriae* (13.2%) were most common isolates for Gram-negative organisms. *Methicillin-resistant Staphylococcus aureus* (*MRSA*) was detected in 2 infants whose symptoms resolved by a routine probing. No difference of bacteriology pattern was detected between patients under 6 months old and those beyond. The pathogens were highly sensitive to chloramphenicol (88%) and levofloxacin (84%), but resistant to erythromycin (40%) and sulfamethoxazole (32%).

**Conclusions:**

Infants with CNLDO under 1 year of age presented predominance of *Streptococcus* as Gram-positive organism, and *Haemophilus* as Gram-negative organism. Levofloxacin was an active topical antibiotic agent with few chance of resistance especially for Chinese children. These findings could help clinicians choose optimal medicine for CNLDO as the conservative treatments.

## Background

Congenital nasolacrimal duct obstruction (CNLDO) is one of the most common causes of tearing in infants, and as many as 11–20% of newborns could be affected by CNLDO [1, 2]. First-line treatment modalities include frequent lacrimal sac (LS) massage and topical antibiotics for the patients passing discharge. If conservative therapy is ineffective, lacrimal duct probing would be advocated. Though controversy exists for the best timing for lacrimal probing [3, 4], conservative treatment in the first year of life was generally recommended due the high spontaneous recovery rate of CNLDO [2, 5, 6].

Thus, it is essential to analyze the microbiology in the microhabitat of LS during the observation period for the patients in their first year of life, because infants have compromised immune system, and misuse of antibiotics could lead to growth of resistant bacteria, a delayed or maltreated CNLDO would lead to severe complications such as acute dacryocystitis, orbital cellulitis, sepsis or meningitis [7].

The previous studies reporting the bacteria strains of CNLDO have a wider age range, or varied ways of sample collection, and some papers are old [8–18]. Few of the studies focused merely on infants under 1 year of age [14, 16], and few looked into the Chinese population [18]. The objective of this study was to analyze the current microbiology spectrum of CNLDO in Chinese children under 1 year old, and to compile a bacterial resistogram which could guide antibiotic regimen.

## Methods

### Study setting, design and population

This study conformed to the provisions of the Declaration of Helsinki and was approved by the Institutional Research Ethics Board of Children’s Hospital Zhejiang University School of Medicine (ethical approval numer: 2020-IRB-087). Children under 1 year old with unilateral CNLDO who received early lacrimal duct probing from May 1, 2017, through August 31, 2018 were included. Patients with punctal or canalicular abnormalities, previous lacrimal duct irrigation, epiphora caused by diseases other than CNLDO and previous usage of systemic antibiotics were excluded.

The medical records of all the subjects were reviewed. Demographic data including the age, sex, residence, and clinical parameters including manifestation of CNLDO, success rates of probing, microbiology culture results, and susceptibility test were collected. Culture results, whether positive or negative, were reviewed and divided into groups according to the strains identified.

### Treatment methods and sample collection

The diagnosis of CNLDO was made in infants with epiphora, increased tear lake and discharge, and confirmed by probing thereafter. All of them had been treated by conservative methods such as Crigler’s LS massage [19]. Levofloxacin, a broad-spectrum antibiotic eye drop, was used if they presented with purulent discharge or any symptoms of ophthalmic infection; if ineffective, tobramycin would be used. No systemic antimicrobial agents were given. Lacrimal duct probing was recommended for the patients who had refractory conjunctivitis that needed continuous use of antibiotics for more than 1 week, patients with severe canthal eczema caused by epiphora, and those whose parents had strong desire for early probing because of their severe anxiety about the babies’ symptoms, poor compliance to LS massage treatment, or inconvenience in follow-up visits.

All the lacrimal probings were done by a single senior ophthalmologist (Dr. XYZ). After local anesthesia, an antiseptic with 5% povidone-iodine was applied to the conjunctival sac, lid margin and periocular skin. A syringe was passed through the upper punctum after punctal dilation, and normal saline was irrigated, aiming to open up the obstruction. A reflux of discharge was collected with cotton swab during syringing. If high-pressure irrigation could not open the nasolacrimal duct, probing was performed with a #6 stainless steel lacrimal probe in the same session.

### Microbiology investigation

The clinical specimens were inoculated into Columbia blood agar and Haemophilus Chocolate agar (BioMérieux, France), and incubated in 5% CO2 for 18–24 h. At the same time, the samples were incubated anaerobically into Columbia blood agar in GENbox anaer (BioMérieux, France). Sabouraud agar (Antu, China) was used for fungal culture. The colonies were identified using Matrix Assisted Laser Desorption Ionization Time of Flight Mass Spectrometry (MALDI-TOF MS, Bruker). Antimicrobial susceptibility test was performed with commercialized microdilution method (VITEK COMPACT, BioMérieux, France) or disk diffusion method, as appropriate, and the isolates were divided into susceptible or non-susceptible (intermediate and resistant) categories according to the breakpoints available from Clinical Laboratory Standards Institute (CLSI) guidelines M100-S28.

### Statistical methods

Statistical analysis was performed using SPSS software, version 20 (SPSS Inc., Chicago, IL). Means and standard deviations were used for continuous variables, rates and percentages were used for categorical variables. Bacteria characteristics according to subjects’ age groups were analyzed with Fisher exact tests. Value of *P* < 0.05 was considered statistically significant.

Subgroup analysis was performed for those under 6 months old vs those beyond 6 months old. The division was made on the basis that stagnant bacteria might reduce the success rate of probing after 6 months of age by inflammatory tissue remodling [20, 21], and that the bacteria might be changed by intermittent use of local antibiotics.

## Results

This study included 32 consecutive otherwise healthy infants with unilateral CNLDO consisting of 23 (71.9%) males and 9 (28.1%) females. Except for 6 patients who did not indicate their residence, 15 (57.7%) children lived in downtown and 11 (42.3%) in countryside. Tearing and purulent discharge were presented in all the patients, who recovered completely after lacrimal probings without developing severe infections such as acute dacryocystitis before or after the procedure. The patients’ age at probing ranged between 1.7 and 12 months, with a mean of 6.7 ± 2.4 months.

Among the 32 collected samples, 28 (87.5%) positive cultures were identified, yielding a total of 38 strains of 19 different bacteria. None of the isolates was anaerobic bacterium or fungus. Sixteen (57.1%) samples yielded only Gram-positive bacteria, 7 (25%) only Gram-negative, and 5 (17.9%) both.

Table 1 summarizes the details of bacterial strains isolated in CNLDO. In 23 (60.5%) Gram-positive strains, *Streptococcus* was the most frequent species (50%), among which *Streptococcus pneumoniae* (*S. pneumoniae*) (23.7%) was predominant, followed by *Streptococcus mitis* (10.5%). *Methicillin-resistant Staphylococcus aureus* (*MRSA*) was detected in 2 (6.3%) infants. In 15 (39.5%) Gram-negative organisms, *Haemophilus species* (21.1%) and *Neisseriae species* (13.2%) were commonly isolated. For the former type, *Haemophilus influenzae* (*H. influenzae*) and *Haemophilus haemolyticus* were often identified, both accounting for 7.9%.

**Table 1:** Bacteriology of children with CNLDO under 1 year of age

Bacteria isolated	Number of isolates	% of isolates (*n* = 38)	% of samples (*n* = 32)
Gram-positive bacteria	23	60.5	65.6
*Streptococci species*	19	50.0	59.4
*Streptococcus pneumoniae*	9	23.7	28.1
*Streptococcus mitis*	4	10.5	12.5
*Streptococcus anginosus*	2	5.3	6.3
*Streptococcus oralis*	1	2.6	3.1
*Streptococcus contellatus*	1	2.6	3.1
*Streptococcus intermedius*	2	5.3	6.3
Others			
*Staphylococcus aureus (MRSA)*	2	5.3	6.3
*Gemella haemolysans*	1	2.6	3.1
*Corynebacterium macginleyi*	1	2.6	3.1
Gram-negative bacteria	15	39.5	37.5
*Haemophilus species*	8	21.1	25.0
*Haemophilus influenzae*	3	7.9	9.4
*Haemophilus parainfluenzae*	1	2.6	3.1
*Haemophilus haemolyticus*	3	7.9	9.4
*Haemophilus aphrophilus*	1	2.6	3.1
*Neisseriae species*	5	13.2	15.6
*Neisseriae flavescens*	1	2.6	3.1
*Neisseriae sicca*	1	2.6	3.1
*Neisseriae elongata*	1	2.6	3.1
*Neisseriae macacae*	2	5.3	6.3
Others			
*Stenotrophomonas maltophilia*	1	2.6	3.1
*Capnocytophaga sputigena*	1	2.6	3.1
Anaerobic organisms	0	0	0
Fungas	0	0	0

Note: *CNLDO* congenital nasolacrimal duct obstruction, *MRSA* methicillin-resistant Staphylococcus aureus

There was no significant difference with respect to the bacteria spectrum between patients under 6 months old and those beyond (Table 2). Ten mixed cultures (31.3%) were identified with 2 bacterial isolates in a single sample. More patients beyond 6 months old had mixed cultures, but no difference of co-colonization rate was found compared to that of patients under 6 months old (*P* = 0.226).

**Table 2:** Comparison of bacteria profile of 2 subgroups demarcated by 6 months old

Age (month)	No. of cases	Positive samples	Mixed cultures	Gram staining			Dominant strains			Resistance to levofloxacin
				Pos	Neg	Pos + Neg	*Streptococci*	*Haemophilus*	*Neisseria*	
< 6	12	11	2	7	3	1	7	2	2	1
> 6	20	17	8	9	4	4	12	6	3	3
*P* value		1.000	0.226	0.758			1.000	0.419	1.000	0.616

Note: *Pos* positive, *Neg* negative. *P* value for Fisher exact tests

Antibiotic susceptibility results were obtained in 22 patients (78.6%). In general, bacteria in CNLDO were most sensitive to chloramphenicol (88%), followed by levofloxacin (84%), ceftriaxone (80%) and vancomycin (76%). *Streptococcus species* exhibited high rates of sensitivity to chloramphenicol (100%), ceftriaxone (94.4%), cefotaxime (94.4%), and vancomycin (94.4%).

**Fig. 1:**
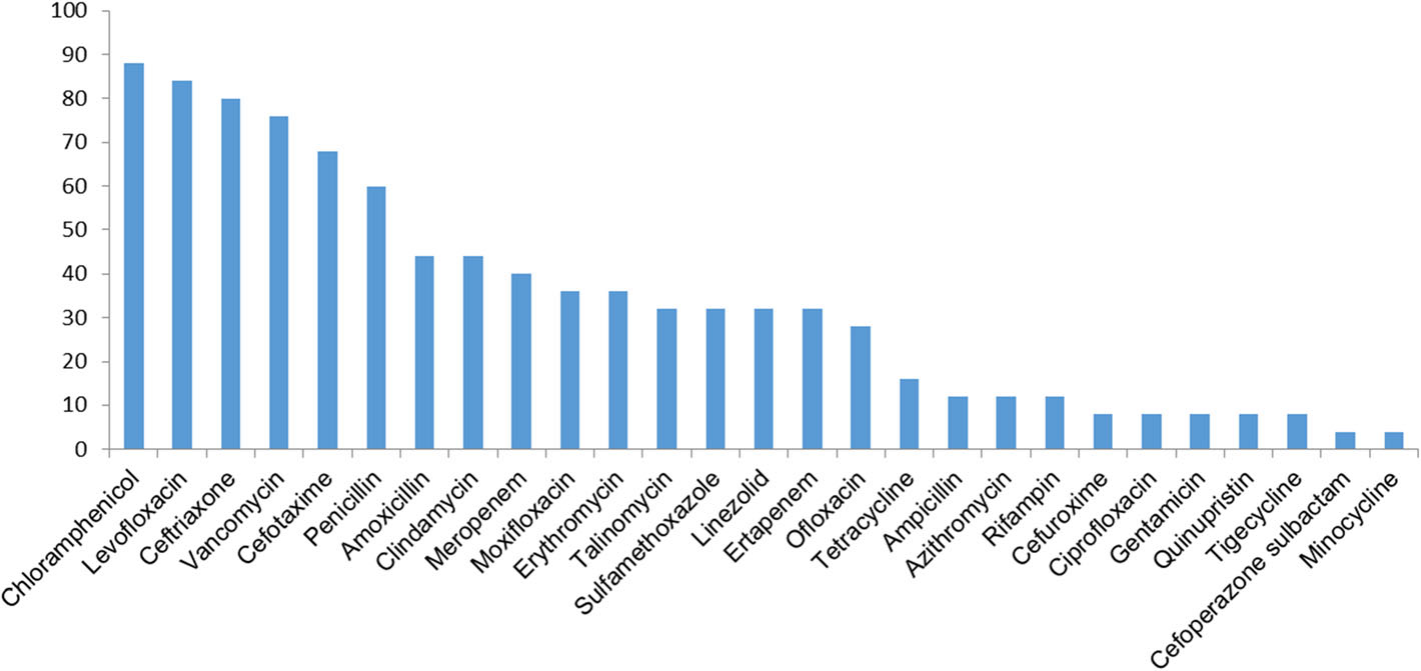
Susceptibility rates of bacterial isolates in CNLDO against different antibiotics

**Fig. 2:**
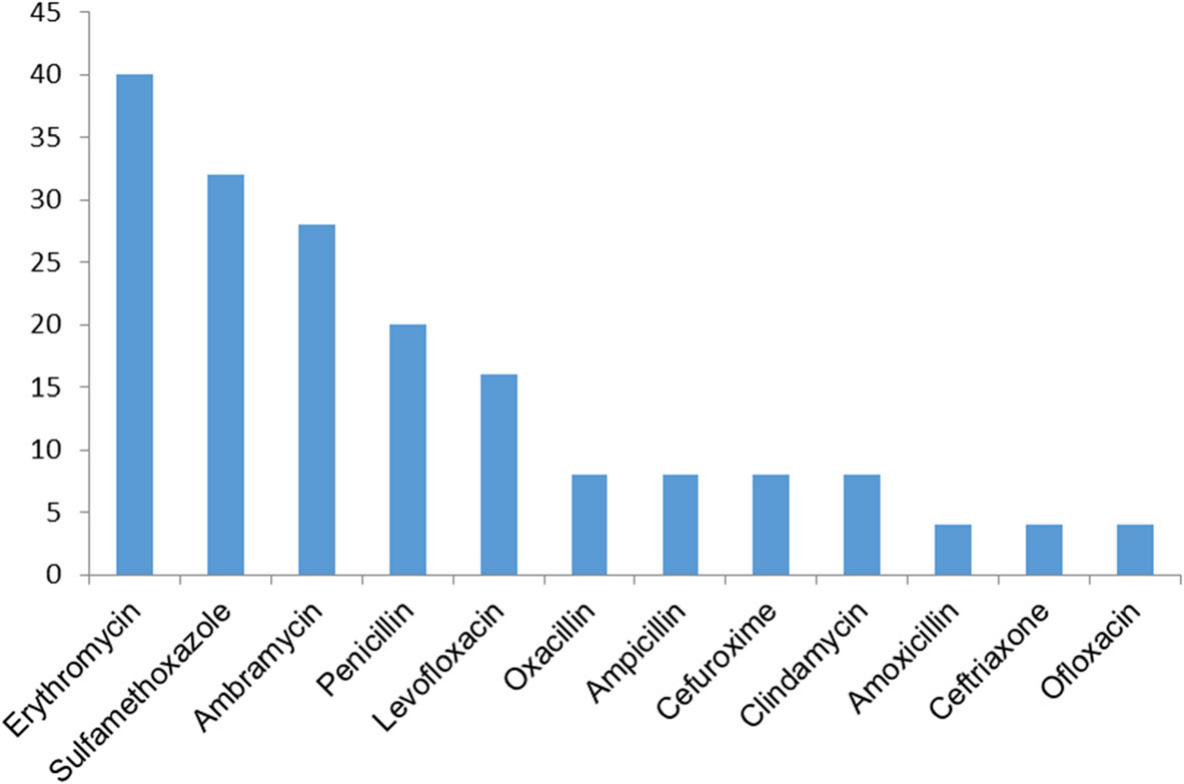
Non-susceptibility rates of bacterial isolates in CNLDO against different antibiotics

Erythromycin and sulfamethoxazole were proven to be ineffective in 40 and 32% of the isolates, respectively. Furthermore, 55.6% of *Streptococcus species* showed resistance towards erythromycin. Levofloxacin, which is the most commonly used topical antibiotics in our practice, exhibited a non-susceptibility rate of 16% in all the bacteria. The sensitivity patterns of microorganisms to antibiotic agents routinely tested are presented in Figs. 1 and 2.

Four patients presented pathogens resistant to levofloxaxin and 3 were beyond 6 months of age. However, the non-susceptibility rate did not differ significantly between infants under and beyond 6 months old (*P* = 0.616), though the older group of patients might use levofloxaxin eye drops intermittently for a longer time during the conservative treatment (Table 2).

## Discussion

Our study was one of the larger studies targeted at children under 1 year old in China. It demonstrated a high positive culture rate in infantile CNLDO. Among all the colonization, *S. pneumoniae* was the major isolate. *Haemophilus* and *Neisseriae species* were also commonly detected colonies. Chloramphenicol and levofloxacin were active agents for most of the pathogens in CNLDO, while erythromycin and sulfamethoxazole were proven to be resisted in a relatively high proportion.

There have been a few studies investigating the microbiology in CNLDO, and the bacterial spectrum varies among different age groups and changes over time (Table 3) [8–11, 13–18]. Most of the previous literatures included both infants and young children. Only 2 studies focused on infants under 1 year old, which were published 2 to 3 decades ago. The latter revealed a prevalent growth of *Staphylococcus aureus* (*S. aureus*) with a positive culture rate of 8.9–25%, and few evidence of *S. pneumoniae* infection (0–2%) [14, 16]. It was inconsistent with our observation, where *S. pneumoniae* constituted for a major proportion of 32.1% and *MRSA* only 7.1%. Though the details of topical antibiotics which the infants used before probing were not mentioned in these 2 literatures, we speculate that this could cause the differences of microorganisms detected in CNLDO [14].

**Table 3:** Review of bacterial pathogen patterns in patients with CNLDO [8–11, 13–18]

Study	Year of study	Country	No. of cases	Age range (mean)	Positive culture rate (%)	Mixed infection rate (%)	Sample collection method	Dominant bacteria (%)	Sensitivity test (%)	Non-susceptibility (%)
Zheng (our study)	2017–2018	China	32	1.7–12 mons (6.6 mons)	87.5	35.7	Irrigation	*Streptococcus pneumoniae* (23.7) *Streptococcus mitis* (10.5) *Haemophilus influenzae* (7.9) *Haemophilus haemolyticus* (7.9) *Neisseriae* (13.2)	Chloramphenicol (88) Levofloxacin (84) Ceftriaxone (80)	Erythromycin (40) Sulfamethoxazole (32)
Bekmez	2017–2018	Turkey	70	12–36 mons (25 mons)	28.6	NA	Conjunctival swab	*Haemophilus haemolyticus* (20) *Haemophilus influenzae* (20)	Ciprofloxacine (85) Tetracycline (60) Gentamicin (40) Penicilin (40)	NA
Prokosch	2007–2012	Germany	132	0–60 mons (27 mons)	97	87	Pressure over LS or irrigation	*Streptococcus pneumoniae* (28.5) *Staphylococcus aureus* (18.3) *Haemophilus influenzae* (8.7) *Moraxella catarrhalis* (8.5)	Ciprofloxacin (99) Chloramphenicol (97) Tobramycin (95) Levofloxacin (95)	Penicillin (65) Erythromycin (41) Gentamicin (22)
Wong	2005–2010	China	16	0–84 mons (26.4mons)	43.7	12.5	Conjunctival scraping	*Streptococcus pneumoniae* (18.8) *Haemophilus influenzae* (12.5)	NA	NA
Kuchar	NA (before 2000)	Austria	47	8–36 mons (21 mons)	70	40	Pressure over LS	*Streptococcus pneumoniae* (36.4) *Haemophilus influenzae* (19.2)	Ofloxacin (84) Tetracycline (84) Chloramphenicol (84) Ciprofloxacin (62) Norfloxacin (60)	Neomycin (63) Gentamycin (54.8) Tobramycin (57.5)
AL-Faky	1998–2008	Saudi Arabia	141	1–108 mons (29.9 mons)	87.9	25.4	Pressure over LS	*Streptococcus pneumoniae* (48.1) *Haemophilus influenzae* (39.2) *Moraxella catarrhalis* (12.7) *Staphylococcus aureus* (6.6)	Bacitracin (NA) Polymyxin B (NA)	NA
Prokosch	2007	Germany	66	6–16 mons	97	87	Irrigation	Gram-pos: *Streptococcus pneumoniae* (31) *Staphylococcus aureus* (13) *Streptococcus epidermidis* (13) Gram-neg: *Branhamella* (12) *Haemophilus influenzae* (11)	Chloramphenicol (99) Fusidic acid (99) Ciprofloxacin (99) Levofloxacin (95) Tobramycin (95)	Erythromycin (41) Gentamycin (22)
Usha	NA (before 2006)	India	187	0–60 mons (14 mons)	83	11.7	Pressure over LS	*Streptococcus pneumoniae* (32.7) *Haemophilus influenzae* (31.3) *Streptococcus viridans* (14.7)	Gram-pos: Chloramphenicol (98) Vancomycin (82) Ofloxacin (75) Gram-neg: Ofloxacin (83) Ciprofloxacin (81)	Gram-pos: Tobramycin (90) Gentamycin (66) Gram-neg: Amikacin (44) Gentamycin (40) Tobramycin (43)
Kim	1996–1999	Korea	50	0.7–12 mons	64	0	Irrigation	*Staphylococcus aureus* (25) *Pseudomonas aeruginosa* (15.6)	Staphylococcus aureus: Vancomycin (100) Ciprofloxacin (87.5) Pseudomonas aeruginosa: Ciprofloxacin (100) Amikacin (100)	Getamicin (NA) Penicillin G (NA)
Bareja	NA (before 1990)	India	87	NA	67.5	0.9	Conjunctival swab with or without pressure over LS	*Streptococcus pneumoniae* (28.9) *Staphylococcus aureus* (13.2)	Cloxacillin (77) Erythromycin (68) Gentamicin (46.9)	Penicilin (100) Streptomycin (97) Polymixin B (98)
MacEwen	1988	UK	158	0–12 mons	19.6	0.6	Conjunctival swab	*Haemophilus influenzae* (12) *Staphylococcus aureus* (8.9) *Moraxella catarrhalis* (2.5)	NA	NA

Note: *LS* lacrimal sac, *Mons* months, *NA* not applicable

Microbiota varies in different microhabitats of human eyes. The ocular surface, conjunctiva, lid margin and skin might show respective distinct bacterial spectra [22]. The sampling location is vital to the analysis of microbiome in CNLDO patients. In most of the previous studies, the samples were obtained from conjunctival discharge by compressing LS. The specimen could be contaminated by the conjunctiva or lid margin, or sometimes little discharge with insufficient bacterial load could be obtained. In our study, we first sterilized the conjunctival sac, palpebral margin and skin, then collected the refluxed secretion from the lacrimal puncta by irrigation. This procedure would ensure maximum amount of specimen from LS.

Up till now, there have been 3 studies collecting irrigation samples of CNLDO, and they were investigated in Germany and Korea more than 10 years ago [9, 14, 15]. These literatures showed a high growth of *S. aureus* (13–25%), a low growth of *Neisseriae* (0.8–2%) and a variable clustering of *S. pneumoniae* (2–31%). The above were in contrast to our study which showed that *Streptococcus* and *Neisseriae* species were the most common, whereas *S. aureus* was a rare isolate. The difference could be attributed to microbiol changes with time, race or locality. Prokosch’s studies were 10 years later than Kim’s, and the former showed prevalence of *S. pneumoniae* which was similar to ours [9, 14, 15]. The Korean children Kim reported were closer to our patients with respect to race (both Asians) and locality compared to those in Prokosch’s studies conducted in Germany, which showed a much fewer isolation of *S. pneumoniae*. Prior antibiotic use might influence the result, and lead to a relatively low positive culture rate (64% vs 87.5% in our study, 97% in Prokosch’s studies) [14]. Non-gonococcal, non-meningococcal *Neisseriae* were rarely reported in CNLDO. Antibiotic sensitivity tests of *Neisseriae* were not routinely performed in our hospital. Whether the bacteria are not susceptible to levofloxacin or tobramycin, or Chinese are genetically more susceptible to *Neisseriae* infection needs further study to prove.

*S. aureus* was one of the most common bacterial pathogens in neonatal conjunctivitis with a positive rate of 17–37.4% about 30 years ago [23, 24]. However, a study from southern China revealed a declining trend of *S. aureus* from 2002 to 2016, which was assumed to be attributed to antibiotics abuse [25]. In our study, we advised patients with conjunctivitis to use levofloxacin every time they passed purulent discharge before probing could be performed, which might lead to no detection of *S. aureus,* and the result was consistent with that of A. Kuchar [8]. So far, *MRSA* has been rarely isolated from infants with CNLDO. Sylvia Kodsi reported a case of *MRSA* cultured from the regurgitated pus in an 8.5-month-old child [26], while the other 2 cases demonstrated its overgrowth in conjunctiva and blood, respectively [27, 28]. MRSA could be related to chronic systemic antibiotics administration [24], recent hospitalization [25] or vertical transmission from the mother [26]. Our study identified 2 infants with *MRSA* colonization in a total of 32 patients. No prior systemic antibiotics were used, no special signs of infection were detected in the infants and their family members, and their ocular symptoms resolved completely after an uneventful probing without causing any other infectious diseases.

*H. influenzae* and *S. pneumoniae* were reported to be prevalent bacteria in CNLDO patients with a wider range of age (Table 3), both pathogens can induce bacteremia after lacrimal probing [29, 30], and *S. pneumoniae* can cause severe endophthalmitis following glaucoma or cataract surgeries [31, 32]. *Neisseriae species* are part of the normal flora in respiratory system. Non-gonococcal, non-meningococcal *Neisseriae* are usually not pathogenic, but they can still lead to severe infections such as sepsis and endocarditis on occasion [33]. Since the high prevalences of *Streptococcus, Haemophilus* and *Neisseriae* were reported in our study, empirical use of antibiotics against these bacteria should be considered as the initial treatment if the infants with CNLDO develop sepsis. Furthermore, the need of investigating the bacteriology after lacrimal probing should be emphasized. By knowing the prevalent bacteria and susceptibility results in CNLDO, it which could help prepare the doctors in treating the potential severe infections especially in young children.

Since bacterial conjunctivitis occurs intermittently in CNLDO, topical antibiotics should be given when a purulent discharge is present [34]. It’s crucial to make prudent choice of antibiotic eye drops for infants, because they might require long term use of eye drops until the infection is treated. Chloramphenicol and levofloxacin were reported to be the most active according to our susceptibility test, and are usually used as topical eye drops instead of systemic antibiotics. Though literatures of infants under 1 year old are uncommon, levofloxacin is still deemed as an effective and safe antibiotic for infants with bacterial conjunctivitis [35, 36]. Despite the activity of chloramphenicol against most bacteria in CNLDO, its ineffectiveness has been reported in pediatric conjunctivitis [37]. Furthermore, it is contraindicated in children with G6PD deficiency, which is common in southern China [38], and some doctors have concern for its possible side effects of gray baby syndrome and aplastic anemia in newborn [39, 40]. It is noteworthy that tobramycin, a commonly prescribed medicine in pediatric clinic [18], may sometimes be ineffective in CNLDO according to literature review (Table 3). Erythromycin, another frequently prescribed ophthalmic prescription [41], is proven to be ineffective for CNLDO, which is in accordance with most of the previous studies regardless of the year of study. Above all, topical administration of levofloxacin would be a better choice for Chinese infantile CNLDO with purulent discharge.

Pollard reported that 2.9% of infants with CNLDO would develop acute dacryocystitis [42], among which, 22.7% were concurrent with bacteremia [43]. In such situation, antibiotics are usually given intravenously, including penicillins, cephalosporins, clindamycin, and vancomycin [7], which are consistent with our study.

The limitations of the study lie in the following aspects: 1. the preceding usage of antibiotics could influence the bacteria profile; 2. our hospital is a tertiary referral institution of CNLDO in Zhejiang which is a well-developed province in southern China. The hospital-based study represent the local condition, but might not represent the other areas in China; 3. the bacteriology of asymptomatic CNLDO might not be included; 4. variation of the bacterial spectrum with age was not certain; 5. retrospective design of the study hindered absolute standardization of intervention and data collection, which in turn reduce the level of evidence. However, the strength of this study is a large sample size focusing only on infants under 1 year old, and the unified treatment strategy and a reasonable way of collecting LS specimen. For the further study, a prospective, multi-centered investigation involving more patients of broader age spectrum with both symptomatic and asymptomatic CNLDO should be conducted.

## Conclusions

The current study shows that the presence of microorganisms is common in infantile CNLDO, and investigating the LS bacteriology after probing is essential. A high rate of isolation of *Streptococcus, Haemophilus* and *Neisseriae species* was found in infants with CNLDO. *MRSA* infection occurred occasionally. With early probing, we got the latest evidence of LS microbial profile in the first year of life. Most doctors would adopt a conservative approach during this period of time, and thus the choice of topical antibiotics for the relatively frequent occurrence of conjunctivitis would be of concern. The present study shows that topical levofloxacin would be a good choice as an empirical treatment during the expectant period, especially in China.

## Supplementary information

**Additional file 1.**

## Data Availability

The datasets used and/or analysed during the current study are available from the corresponding author on reasonable request.

## Abbreviations

CLSI: Clinical Laboratory Standards Institute
CNLDO: Congenital nasolacrimal duct obstruction
*H. influenzae*: *Haemophilus influenzae*
LS: Lacrimal sac
*MRSA*: *Methicillin-resistant Staphylococcus aureus*
*S. aureus*: *Staphylococcus aureus*
*S. pneumoniae*: *Streptococcus pneumoniae*
